# Targeting the LPS-STING axis: neomycin restores STING-mediated anti-tumor immune suppression and inhibits tumor growth

**DOI:** 10.3389/fimmu.2025.1637667

**Published:** 2025-10-29

**Authors:** Hong Fan, Dongjie Fu, Mingfu Tian, Zhiqiang Li, Siyu Liu, Chenglin Ye, Kailang Wu, Chengliang Zhu

**Affiliations:** ^1^ Department of Clinical Laboratory, Institute of Translational Medicine, Renmin Hospital of Wuhan University, Wuhan, Hubei, China; ^2^ Department of Stomatology, Renmin Hospital of Wuhan University, Wuhan, China; ^3^ State Key Laboratory of Virology, College of Life Sciences, Wuhan University, Wuhan, Hubei, China

**Keywords:** microbiome, lipopolysaccharide, TBK1, tumor, neomycizn

## Abstract

**Introduction:**

The interplay between microbial metabolites and host immunity within the tumor microenvironment (TME) critically modulates anti-tumor immune responses. The role of Gram-negative bacteria and their cell wall component lipopolysaccharide (LPS) in this context warrants further investigation.

**Methods:**

We assessed the impact of low-dose LPS pretreatment on macrophage function by measuring type I interferon (IFN-β) secretion in response to tumor cell debris. Mechanistic insights were gained by analyzing endogenous signaling pathways in macrophages. The therapeutic potential of targeting LPS was evaluated in melanoma-bearing mice treated with neomycin, alone or in combination with STING agonists.

**Results:**

Low-dose LPS pretreatment significantly suppressed IFN-β secretion by macrophages, indicating LPS-mediated immunosuppression. Mechanistically, LPS disrupted endogenous signaling pathways, blunting the ability of macrophages to sense tumor-derived damage signals. *In vivo*, neomycin treatment markedly inhibited melanoma growth and synergized with STING agonists.

**Discussion:**

Our findings demonstrate that elevated LPS in the TME inhibits anti-tumor innate immunity by impairing macrophage function. The combination of LPS modulation via neomycin with innate immune activation via STING agonists presents a potential strategy to enhance tumor immunotherapy.

## 1 Introduction

Tumor development is a complex biological process involving multiple factors. It is not solely driven by genetic and epigenetic alterations in tumor cells but is also profoundly shaped by the tumor microenvironment (TME) (1–3). Recent advances in microbiomics have revealed that the microbiota is a critical component of the TME, highlighting its significance and the need for further investigation (4, 5). This progress stems largely from high-throughput sequencing technology, which has identified characteristic microbial colonization in tumor tissues—previously considered sterile (6). These microbes have been observed to coexist with their hosts for extended periods, thereby establishing intricate interactions that have the potential to exert a profound influence on the biological behaviour of tumors (7, 8).

Conventionally, cancer research and microbiology have been regarded as discrete domains. However, the identification of bacteria in tumor tissue has prompted scientists to re-evaluate the potential association between bacteria and tumours (5). Recent research findings indicate a potential role for bacteria in the development, progression and metastasis of tumors (9–12). These microorganisms that colonize tumor tissues are collectively termed the ‘Intratumoral Microbiota’ (IMM) (13). Despite the fact that a significant number of studies have investigated the effects of bacteria on tumor progression and treatment response, the precise mechanism of action remains to be elucidated, particularly with regard to the role of microbes in shaping the tumor microenvironment through metabolites or immunomodulation. This aspect requires further investigation (14).

Bacteria, as key components of the TME, produce a wide range of genotoxic and metabolically active substances, which promote tumorigenesis and progression through diverse molecular mechanisms (15, 16). Among these bacterial components, 16S rRNA and LPS exhibit distinctive biological properties and are detectable in nearly all tumor types, showing highly conserved spatial distribution patterns. This widespread presence suggests that LPS may play a fundamental role in tumorigenesis. In contrast, lipoteichoic acid (LTA), a surface component of Gram-positive bacteria, is rarely detected in most tumor tissues (5, 17, 18). The selective enrichment of LPS (or Gram-negative bacteria) further highlights their unique function in tumor development (19).

Under normal conditions, LPS activates the NF-κB signaling pathway in immune cells such as macrophages and dendritic cells (DCs), stimulating their secretion of pro-inflammatory cytokines and immunomodulatory factors to promote anti-tumor immune responses (20). However, within the tumor microenvironment (TME), the sustained NF-κB activation driven by chronic LPS exposure becomes a hallmark of cancer-associated inflammation and exerts an opposing effect. This persistent signaling reprograms macrophages towards a pro-tumoral, M2-like state, which in turn enhances tumor cell proliferation, survival, migration, and invasion (21, 22). This suggests that LPS-induced macrophage immune tolerance may underlie this paradoxical effect.

A thorough investigation of LPS mechanisms in the TME may yield dual benefits: advancing our understanding of tumor-microbiome interactions at the molecular level and informing the design of innovative anticancer therapies. This article explores the emerging role of intratumoral bacteria in oncogenesis, with a focus on deciphering how LPS modulates immunity within the TME. Our findings aim to provide mechanistic insights for developing next-generation microbiome-targeted anticancer strategies.

## 2 Materials and methods

### 2.1 Mice

C57BL/6J mice were obtained from Hubei Provincial Laboratory Animal Center (Wuhan, China). All animal experiments were conducted in strict adherence to the Animal Welfare Act and the Guide for the Care and Use of Laboratory Animals, in accordance with the procedures approved by the IACUC of the State Key Laboratory of Virology, College of Life Sciences, Wuhan University. All mice were reared under specific pathogen-free conditions within the animal facility of the College of Life Sciences, Wuhan University. Male mice aged 6–8 weeks were used in all experiments.

### 2.2 Isolation of peritoneal macrophages from mice

Four days before the experiment, 1 mL of 3% sodium thioglycolate(108191, Millipore) was injected(i.p.) to each C57BL/6J WT mice to induce the aggregation of peritoneal macrophages. The mice were euthanised by cervical dislocation and subsequently fixed in the supine position on an anatomical plate. The abdominal skin was sterilised with 75% alcohol. Using sterile scissors and forceps to lift the abdominal skin, a small incision was made to avoid damage to the abdominal viscera, and 7 mL of phosphate-buffered saline (PBS) was injected into the abdominal cavity. The abdomen of the mice was gently massaged for 2–3 minutes to allow the intraperitoneal fluid to be thoroughly mixed, and then the peritoneal lavage fluid was withdrawn with a sterile syringe and collected into a centrifuge tube. The collected fluid was centrifuged at 300×g for 5 min, and the supernatant was discarded to obtain peritoneal macrophage precipitates.

### 2.3 Cell culture

Mouse colon cancer cell line MC38 and mouse melanoma cell line B16 were obtained from the American Type Culture Collection (ATCC). Cells were cultured in DMEM high glucose medium (Gibco-ThermoFisher) supplemented with 10% FBS and 1% penicillin-streptomycin solution and grown at 37°C in a humidified 5% CO2 incubator.

### 2.4 RNA isolation and quantitative real-time PCR

The cells was first homogenized with 500 μL TRIzol™ Reagent (Invitrogen), 100ul chloroform is introduced and vortexed, then centrifuged at 12000 rpm for 5 min causing the homogenate to segregate into distinct layers: a clear upper aqueous layer that harbors RNA, an intermediate interphase, and a red lower organic layer which contains DNA and proteins. To obtain RNA, 200ul isopropanol is utilized to precipitate it from the aqueous layer and the precipitate was finally washed with 500ul 70% ethanol. RNase-free EP tubes are required for the entire procedure. Using Hiscript II Q RT SuperMix (Vazyme Biotech, China) for cDNA synthesis in the 96-well Veriti Thermal Cycler (Thermo Scientific, China).

### 2.5 Western blotting

Protein samples were mixed with 2×SDS loading buffer, heated at 95°C for 10 min, and then separated by 10% SDS-PAGE. Proteins were transferred onto PVDF membranes, which were blocked with 5% nonfat milk and incubated overnight at 4°C with primary antibodies. The following antibodies were used: anti-phospho-TBK1 (ab109272, Abcam), anti-phospho-TAK1 (ab109404, Abcam), anti-TBK1 (ab40676, Abcam), anti-TAK1 (ab09526, Abcam), and anti-GAPDH (G9295, Sigma). The next day, membranes were incubated with HRP-conjugated secondary antibodies (Jackson ImmunoResearch, USA) for 1 h. After washing, protein bands were visualized using an ECL kit (E423-02, Vazyme Biotech).

### 2.6 Measurement of serum LPS level

Whole blood samples collected from mice were left at room temperature for 2 h and centrifuged at 1000×g for 20 min to separate the serum. LPS levels in mouse serum were measured using a commercial ELISA kit (JL20691, Jianglai Biotechnology).

### 2.7 Cell viability

A cell suspension was prepared and 10^4^ cells were seeded into each well of a 96-well plate. After cell adhesion, cells were treated with LPS at varying concentrations for 24 h, after which 10 μl of CCK-8 solution (CCK004, Biolight Biotechnology) was added to each well. Plates were incubated for 1–4 h, and absorbance was measured at 450 nm.

### 2.8 DNA extraction

After cell collection, 20 µL proteinase K and 300 µL PK buffer (10 mM Tris-HCl, pH 8.0) were added to the samples, followed by incubation at 56°C for 1 h in a water bath with gentle inversion every 10 min. When the solution turned clear, 100 µL phenol-chloroform (p1013; Polarbio) was added, and the mixture was centrifuged at 12, 000 ×g for 5 min at room temperature. The upper aqueous phase (200 µL) was carefully transferred and mixed with an equal volume of isopropanol. After repeating the centrifugation, the pellet was washed with 500 µL of 75% ethanol.

### 2.9 Immunofluorescence

Mouse tissues were collected, washed with PBS, and fixed in 4% paraformaldehyde. After paraffin embedding, tissue sections were prepared and subjected to immunohistochemical staining using the following primary antibodies: anti-phospho-STING (Ser366; 19851-1-AP, Proteintech) and anti-LPS (ab35654, Abcam). Nuclei were counterstained with DAPI.

### 2.10 Statistical analysis

All statistical analyses in this study were performed using GraphPad Prism (version 10.1.2). The results were displayed as mean ± SEM. The statistical significance of the differences between the groups was evaluated using the Student’s t-test, and p-value <0.05 was considered statistically significant.

### 2.11 Establishment of tumor-implantation mice model

WT C57BL/6J mice were subcutaneously injected with 200 μL of B16-F10 cells (1.0×10^5^ cells/mouse) suspended in sterile PBS. Three days later, tumor-bearing mice were randomly divided into three groups (n=4/group): B16 (PBS control), LPS (1 mg/kg, L2630, Sigma-Aldrich), and E. coli (1×10^8^ CFU, CD201, TransGen Biotech). Starting on day 4, the LPS and E. coli groups received intraperitoneal injections of their respective treatments on alternating days. Tumor length (a) and width (b) were measured every 3 days, and volume (V) was calculated as V = ½ × a × b². When V reached 1500 mm³, tumors were excised entirely. One portion was fixed in 4% paraformaldehyde for 24 h, and the remainder was snap-frozen in liquid nitrogen for molecular analysis (23–25).

### 2.12 *In vivo* assays for neomycin

WT C57BL/6J mice were subcutaneously inoculated with B16-F10 melanoma cells (1.0×10^5^ cells/mouse) to establish the tumor model. Three days post-inoculation, tumor-bearing mice were randomly assigned into four groups (n=4/group) (1): PBS control (2); Neomycin (30 mg/kg) (3); CMA (Cridanimod; 1.5 mg/kg, T5317, TargetMol); and (4) Neomycin + CMA combination. Treatments were administered intraperitoneally on alternating days.

## 3 Results

### 3.1 LPS enrichment in the tumor microenvironment

**Figure 1:**
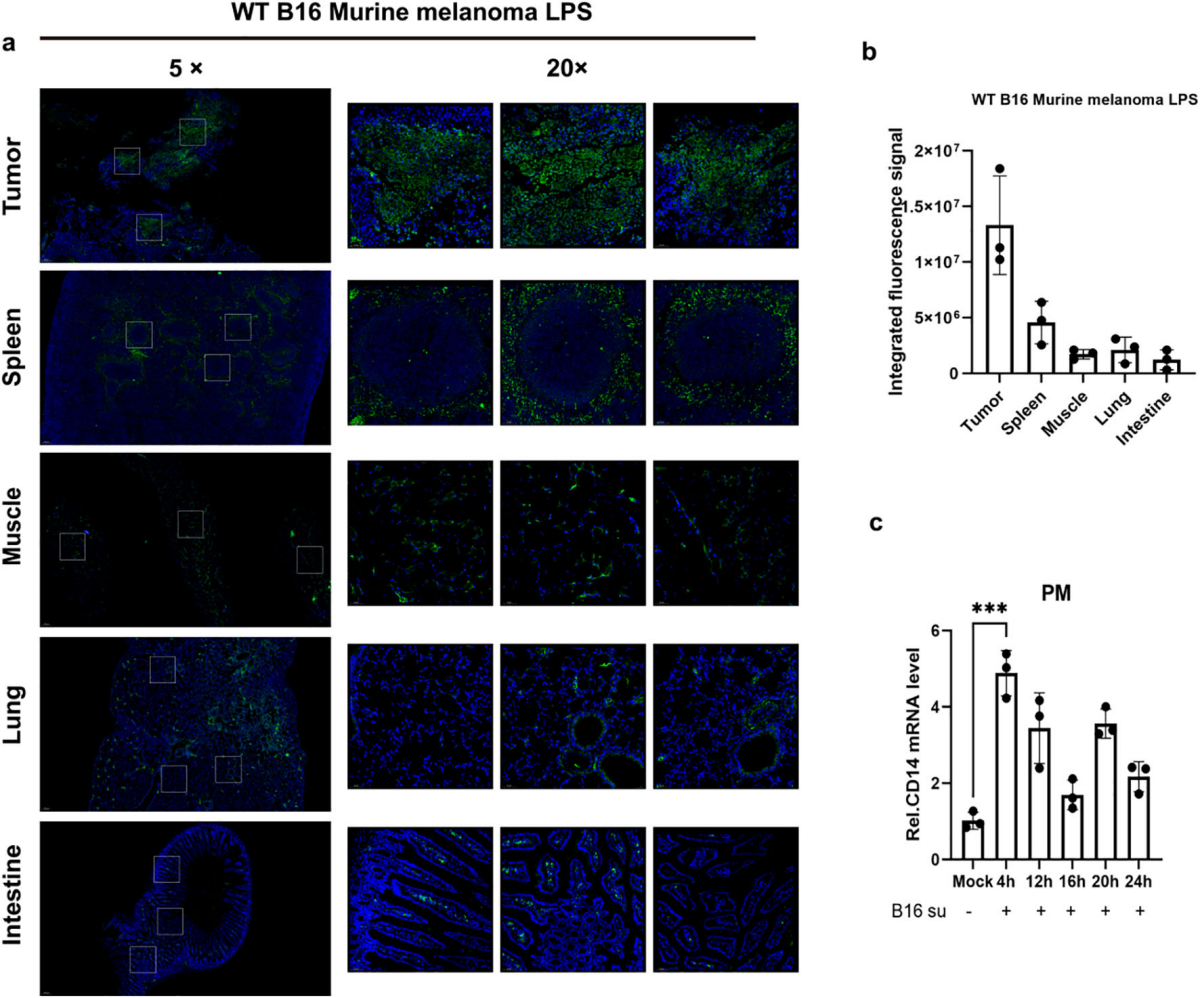
Detection of LPS enrichment within the tumor microenvironment. **(A)** Immunofluorescence staining of LPS was performed in tumor tissues and major solid organs of melanoma tumor-bearing mice (C57BL/6J, n = 4). LPS was labeled in green and nuclei were counterstained with DAPI (blue). **(B)** Quantitative analysis of the fluorescence results was conducted, and the data were presented as mean ± standard deviation. **(C)** Primary mouse peritoneal macrophages were incubated with 50% (v/v) B16-conditioned medium (B16-CM) (collected after 48 h of culture in serum-free DMEM) for 4, 12, 16, 20, or 24 h Control groups received fresh complete medium under identical conditions. Cd14 mRNA levels were quantified by qPCR. ***P < 0.001.

To explore the distribution characteristics and potential functions of LPS in the tumor microenvironment, we first established a subcutaneous xenograft model of B16 melanoma in wild-type C57BL/6J mice. When the tumors reached 500 mm³, tumor tissues, adjacent muscle tissues, and major solid organs (including the liver, spleen, and lungs) were systematically collected. Through immunofluorescence staining combined with quantitative image analysis, we found that the signal intensity of LPS in tumor tissues was significantly higher than that in other normal tissues (**Figures 1A, B**), suggesting that there might be a unique LPS enrichment mechanism in the tumor microenvironment. Notably, *in vitro* experiments showed that the conditioned medium of B16 cells could significantly up-regulate the expression level of Cd14 mRNA in mice peritoneal macrophages (**Figure 1C**). Based on these findings, we speculate that the LPS enriched in the tumor might affect tumor exerts its influence on tumor progression through specific mechanisms.

### 3.2 Gram-negative bacteria promote tumor growth through LPS

**Figure 2:**
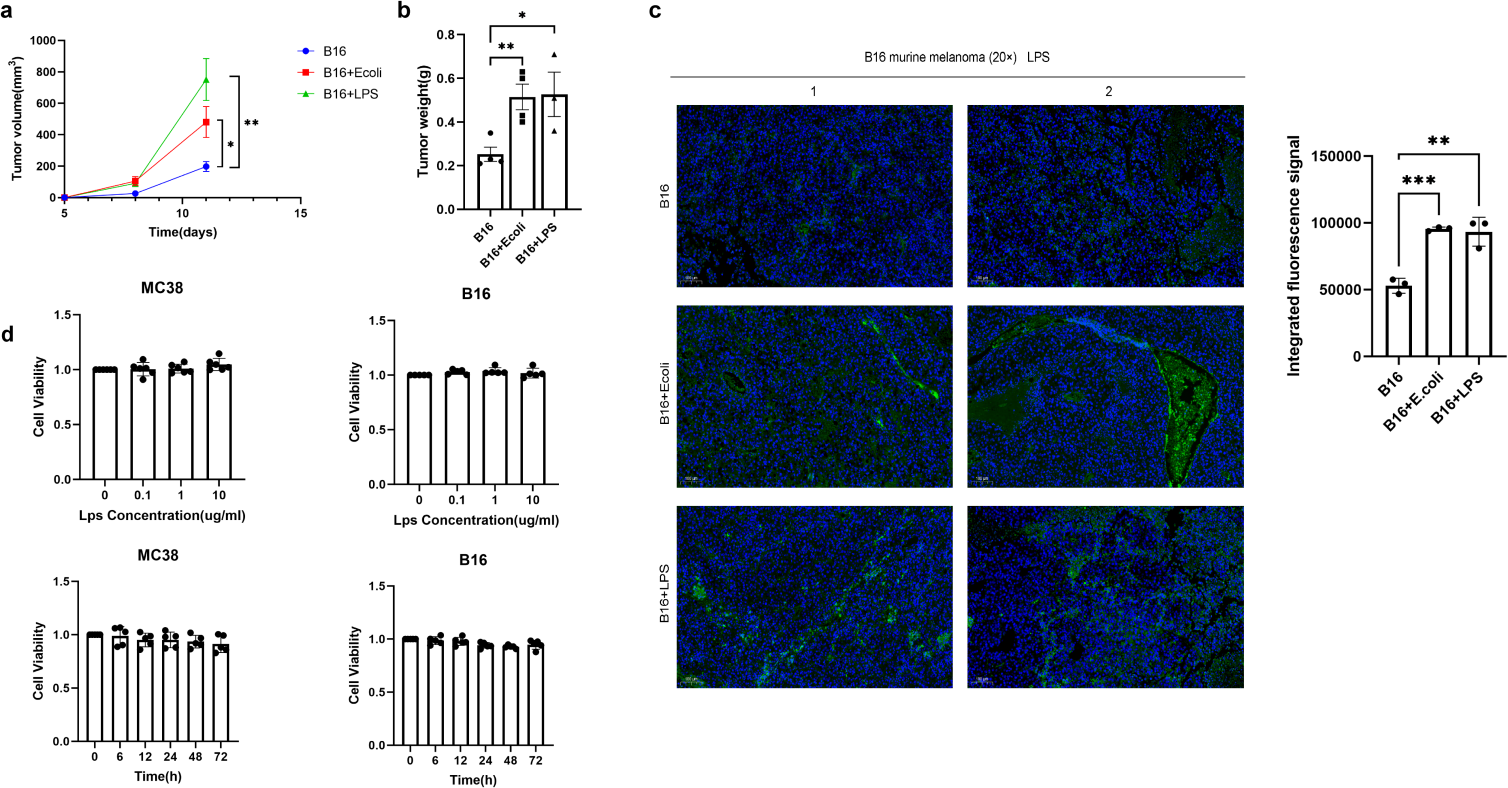
Gram-negative bacteria drive tumor progression via LPS-induced immunosuppression. **(A)** Melanoma tumor-bearing mice (C57BL/6, n=4/group) received intratumoral injections of PBS (control), inactivated E coli (1×10^7^ CFU), or LPS (1 mg/kg) every two days. Tumor volume was calculated by vernier caliper measurement (volume=length×width²×0.5), and data are presented as mean ± SEM. **(B)** Comparison of tumor weight at the end point. **(C)** Immunofluorescence staining of LPS was performed using specific markers to evaluate the abundance of LPS in tumor tissues among the Control group, E coli group and LPS group samples. LPS was labeled green and DAPI nuclear staining was blue. Data are shown as mean ± SD. **(D)** MC38 and B16-F10 cells were treated with different concentrations of LPS (0.1, 1, 10 μg/mL) for 6, 24, 48, and 72 hours. Cell viability (OD450 nm) was measured by CCK-8 assay at each time point. Data were normalized to 100% activity of the untreated group at the corresponding time point and expressed as mean ± SD (n = 3). Statistical analysis showed no significant cytotoxicity of LPS in either cell line across all tested conditions. *P < 0.05, **P < 0.01, and ***P < 0.001.

We sought to determine the contribution of Escherichia coli (E. coli) to tumor progression. In mice bearing B16F10 melanoma, administration of inactivated E. coli or its purified LPS component both enhanced tumor growth relative to controls. A key observation was that the LPS-treated group displayed a steeper tumor growth curve (**Figure 2A**), indicating a faster growth rate, despite the lack of a significant difference in final tumor mass between the E. coli and LPS groups (**Figure 2B**). This kinetic profile implies that the potent immunomodulatory molecule LPS is a primary driver of this effect. The findings support a model wherein the tumor-promoting capacity of Gram-negative bacteria can be largely attributed to LPS, suggesting that live bacteria are not obligatory for this process.

The bioactive components derived from bacteria, such as lipopolysaccharide or other metabolites, seem sufficient to sustain the process of promoting tumor growth. Immunofluorescence analysis revealed substantial LPS accumulation in tumors after E. coli or LPS administration (**Figure 2C**). While LPS did not affect tumor cell proliferation *in vitro* (**Figure 2D**), its *in vivo* effects likely occurby modulating tumor-associated macrophages (TAMs) and inducing immunosuppressive cytokines, suggesting microenvironment-mediated promotion. These findings establish LPS as a key mediator of bacteria-driven tumor progression, acting primarily through microenvironmental regulation rather than direct tumor cell stimulation.

### 3.3 LPS suppresses STING-mediated anti-tumor immunity

**Figure 3:**
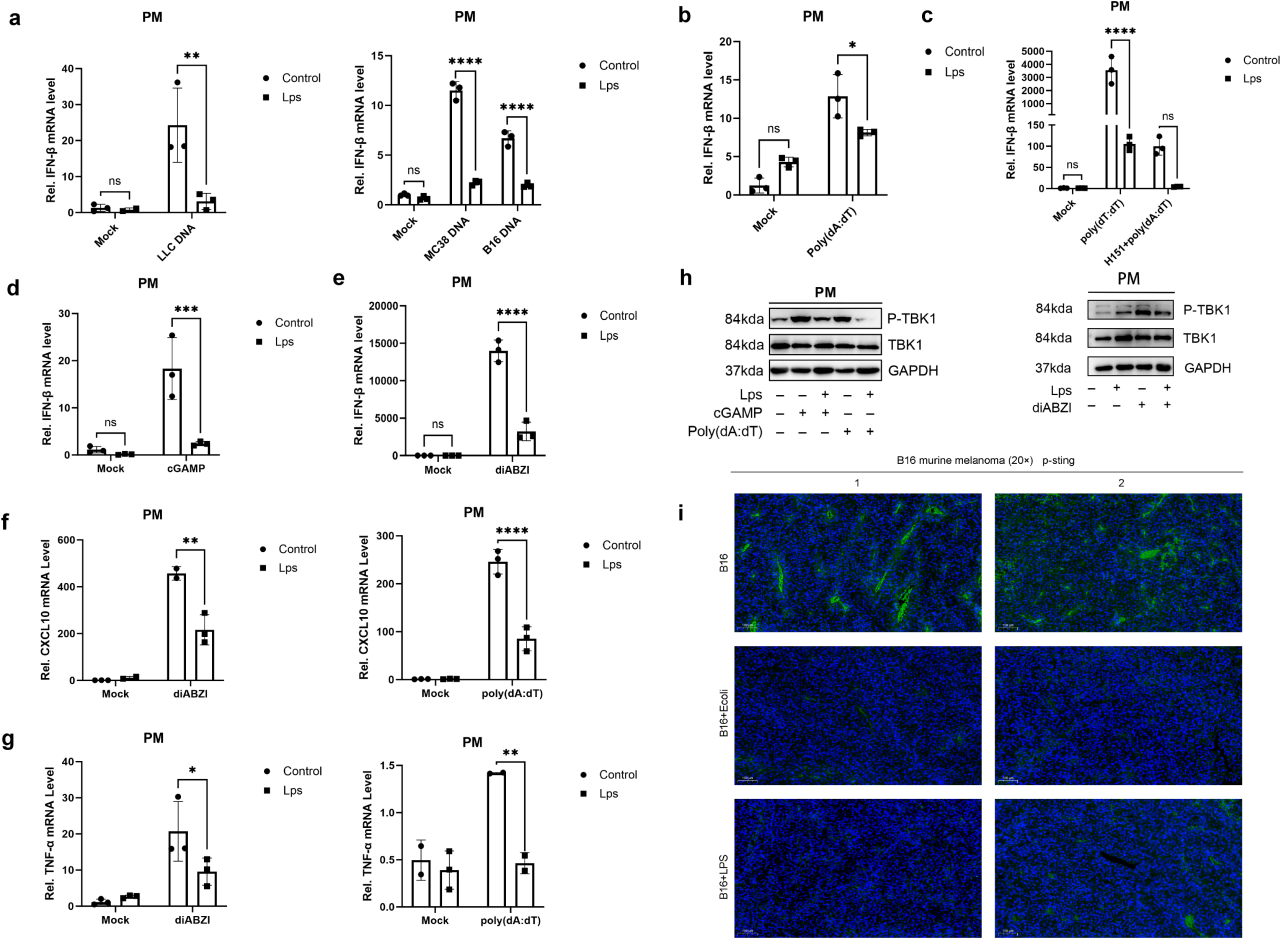
LPS impairs STING-dependent anti-tumor immunity through suppression of cytokine and chemokine production. **(A)** qRT-PCR showed the relative IFN-β mRNA expression in untreated mice peritoneal macrophages (Control group, n = 3) and LPS-pretreated mice peritoneal macrophages (LPS group, n = 3) stimulated with tumor-derived DNA. Data are shown as mean ± SD. (b, d&e) Mouse peritoneal macrophages (PMs) were pretreated with lipopolysaccharide for 24 hours and then washed twice with PBS. PM cells were then stimulated with poly(dA:dT) **(B)**, cGAMP **(D)** or diABZI **(E)**, respectively. The mRNA levels of IFN-β were detected by qRT-PCR. **(C)** IFN-β mRNA was measured by pre-treatment with LPS for 24 h, with H151, an inhibitor of the STING signalling pathway, for 1 h, and by poly(dA:dT) transfection (6–8 h). (f&g) Mouse peritoneal macrophages (PMs) were pretreated with lipopolysaccharide for 24 hours and then washed twice with PBS. PM cells were then stimulated with poly(dA:dT) or diABZI, respectively. The mRNA levels of **(F)** CXCL10 and **(G)** TNF-α were detected by qRT-PCR. **(H)** Under the conditions with or without LPS pretreatment, mouse peritoneal macrophages were stimulated with poly(dA:dT) (6–8 h) or cGAMP (4–6 h) via polyethyleneimine (PEI) transfection at a 2:1 mass ratio of PEI to nucleic acids, or with the STING agonist diABZI (4–6 h). The phosphorylation and total protein levels of TANK-binding kinase 1 (TBK1), including phospho-TBK1 (Ser172), were analyzed by Western Blot with GAPDH as the loading control **(I)** Immunofluorescence Detection of Phosphorylated STING (indicated by green) in Tumor Tissues, with DAPI (in blue) serving as the nuclear counterstain. The magnification is denoted by a 100μm scale bar. Data are expressed as mean ± SD of three independent experiments, two-way ANOVA. *P < 0.05, **P < 0.01, ***P < 0.001, and ****P < 0.0001.

Subsequently, we extracted the DNA from a variety of tumor cells, including LLC, MC38, and B16. Macrophages were pretreated with LPS, and then stimulated with the tumor cell DNA. Intriguingly, it was observed that upon LPS pretreatment, the macrophages failed to produce a high level of IFN-β in response to the tumor DNA stimulation (**Figure 3A**). This finding strongly suggests that LPS is capable of suppressing the anti-tumor immune response. In our study, macrophages were pretreated with LPS, and subsequently, the stimulant was replaced with nucleic acid analogues. These nucleic acid analogues are capable of activating macrophages to produce IFN-β through different receptors. Among the two nucleic acid analogues examined, LPS exhibited an inhibitory effect on the IFN-β production in macrophages induced by the double-stranded DNA analogue poly(dA:dT) (**Figures 3B, C**). Consistent with the suppression of IFN-β, the production of key immune mediators CXCL10 (**Figure 3F**) and TNF-α (**Figure 3G**) was also significantly attenuated by LPS pretreatment, indicating a broad suppression of innate immune activation.

Within cells, the major DNA sensor cGAS activates the crucial cGAS-STING pathway upon DNA recognition. Notably, cGAS can acutely sense tumor-derived DNA, thereby triggering an anti-tumor immune response. This characteristic provides an important theoretical basis for subsequent research. To further investigate this process, we separately used the second messenger cGAMP and the STING-specific stimulator diABZI to stimulate macrophages. As a result, it was found that the production of IFN-β in macrophages was also inhibited (**Figures 3D, E**). Similarly, the induction of both CXCL10 and TNF-α by these STING agonists was markedly suppressed in LPS-pretreated macrophages (**Figures 3F, G**). This phenomenon fully indicates that the regulation of IFN-β production in macrophages by lipopolysaccharide is not isolated but shows obvious pathway dependence, that is, it depends on specific cell signal transduction pathways to achieve its regulatory effect.

To elucidate the molecular mechanism underlying this suppression, we examined the activation of key signaling components. Western Blot analysis revealed that LPS pretreatment significantly inhibited the phosphorylation of TANK-binding kinase 1 (TBK1) at Ser172 in macrophages stimulated with poly(dA:dT), cGAMP, or diABZI, while total TBK1 levels remained unchanged (**Figures 3H**). This suppression of TBK1 phosphorylation provides mechanistic evidence for the observed inhibition of downstream cytokine production. To further verify the actual situation of this conclusion *in vivo*, we established a melanoma mouse model. In this model, we compared the data of the E. coli group/LPS group with that of the control group. The experimental results showed that compared with the control group, the phosphorylation level of STING in the E. coli group/LPS group was significantly reduced (**Figure 3I**). This *in vivo* experimental result is highly consistent with our previous findings at the cellular level, further confirming the close connection between the regulation of IFN-β production in macrophages by LPS and the STING pathway.

### 3.4 Neomycin suppresses tumor growth by targeting LPS

**Figure 4:**
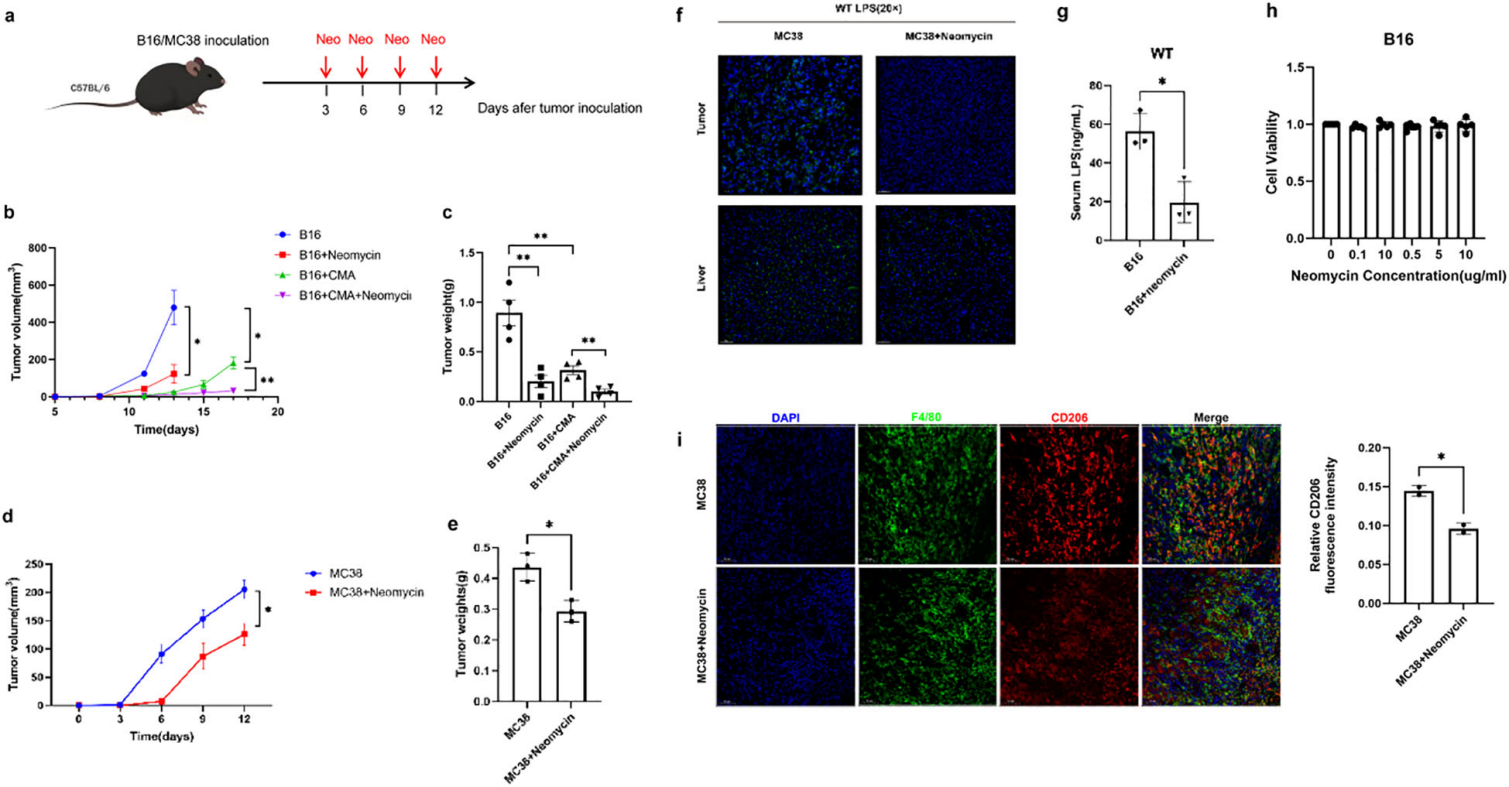
Neomycin inhibits tumor progression through targeting LPS and remodeling the immunosuppressive microenvironment. **(A)** Schematic representation of treatment model. The growth curve **(B)** and weight **(C)** of mice melanoma treated with 30 mg/kg neomycin, 1.5 mg/kg CMA, and their combination were compared with the control group. Comparisons were carried out at specific time intervals, the growth curve **(D)** and the weight **(E)** of colon carcinomas in mice treated with 30 mg/kg neomycin in contrast to the control group. **(F)** Immunofluorescence assays were performed to visualise LPS in both tumor and liver tissues. As shown in the figure, the left panel represents the control group, while the right panel corresponds to the treatment group. DAPI (shown in blue) was used as a nuclear counterstain. Magnification is indicated by a 50 μm scale bar. **(G)** The graph presents the quantified levels of LPS in mice serum samples. The x-axis indicates different experimental groups, which include the control group (B16) and the treatment group (B16+neomycin). **(H)** B16-F10 cells were treated with different concentrations of Neomycin (0.1, 0.5, 1, 5, 10 μg/mL) for 72 hours. Cell viability (OD450 nm) was measured by CCK-8 assay at each time point. **(I)** Representative immunofluorescence micrographs of MC38 tumor sections from control and neomycin-treated mice. Tissue sections were immunostained for the pan-macrophage marker F4/80 (red) and the M2-like polarization marker CD206 (green), with nuclear counterstaining by DAPI (blue). Scale bar: 50 μm. There are three replicates in each group, the error bars reflect the spread of three measurements. Data are shown as mean ± SD. Statistical significance was indicated by * with P < 0.05, ** with P < 0.01.

Next, we focused our attention on the potential role of neomycin, a drug targeting Gram-negative bacteria, in tumor treatment. For this purpose, we selected two representative mouse tumor models, melanoma and colon cancer. During the experiment, the mice in the treatment group received precise neomycin treatment intervention, while the control group did not receive any special drug treatment (**Figure 4A**). In the mice treated with neomycin, the growth rate of the tumor was significantly slowed down, the increase in tumor size was significantly lower than that in the control group, and the tumor weight was also relatively lighter (**Figures 4B–E**). This significant difference indicates that neomycin has played an active and effective role in inhibiting the development of the tumor, strongly proving its potential anti-tumor activity. In order to further explore the internal mechanism of neomycin’s anti-tumor effect, we further carried out detailed analysis and detection on the serum and the components inside the tumor of the mice. The results showed that in the mice treated with neomycin, the LPS content inside the tumor tissue showed an obvious decreasing trend (**Figure 4F**). At the same time, the LPS content in the serum was also significantly reduced compared with that in the control group (**Figure 4G**). Notably, this anti-tumor effect was not due to a direct cytotoxic effect of neomycin on cancer cells, as in vitro CCK-8 assays demonstrated that neomycin treatment did not directly promote tumor cell proliferation (**Figure 4H**). Importantly, immunofluorescence analysis revealed that neomycin treatment substantially reduced the infiltration of CD206^+^ M2-like macrophages in the tumor microenvironment (**Figure 4I**), indicating a reversal of the immunosuppressive state. These findings collectively reveal the mechanistic pathway of neomycin’s anti-tumor action: by reducing LPS content, neomycin remodels the tumor microenvironment and modulates immune regulatory pathways, particularly by shifting macrophage polarization away from the M2-like phenotype, ultimately achieving effective suppression of tumor development.

## 4 Discussion

Abnormal accumulation of LPS in the tumor microenvironment (TME) has become an important phenomenon studied in recent years, and our study further validates this important finding with experimental data (26, 27). This accumulation may originate from two main pathways. Firstly, there is the concept of gut microbiota translocation, whereby the integrity of the intestinal barrier is compromised, resulting in the intestinal microbiota breaching the intestinal mucosal barrier and migrating to distal organs via the portal vein system or the lymphatic circulation. Secondly, the specific colonization and proliferation of tumor-associated bacteria (such as Fusobacterium nucleatum or Escherichia coli) in tumor tissues, which continuously release LPS to form a local high-concentration microenvironment (28, 29). To elucidate the functional impact of this LPS enrichment, we employed both E. coli and purified LPS in our experimental systems. While E. coli modeled the complex biological scenario of bacterial encounter within the TME, purified LPS allowed us to precisely attribute the observed immunosuppressive effects—specifically the suppression of STING signaling and reduction in IFN-β production—to this key. Future studies directly comparing the effects of whole bacteria versus purified PAMPs on this pathway will provide further mechanistic insights. However, the aberrant accumulation of such LPS in the tumour microenvironment, caused by either approach, has a promoting effect on tumour growth, and the mechanisms behind it are extremely complex and multifaceted (5, 30–32).

Extensive research has established that LPS can directly enhance tumor cell proliferation and migration through activation of canonical signaling pathways such as TLR4/NF-κB (33–35). Importantly, the pro-tumorigenic role of LPS/TLR4/NF-κB signaling extends beyond colorectal cancer, with demonstrated significance in bone cancer, hepatocellular carcinoma, and ovarian cancer, highlighting its broad relevance as a therapeutic target across malignancies (36–38). However, our study uncovers a unique indirect pro-tumorigenic mechanism of LPS in colorectal cancer MC38 cell lines. Contrary to conventional understanding, our experimental findings demonstrate that LPS does not directly stimulate MC38 cell proliferation, but indirectly drives tumor progression by remodeling the phenotype and function of TAMs in the TME. This discovery is highly consistent with recent studies on the pancreatic cancer microenvironment, which elucidated that LPS regulates macrophage polarization in a time-dependent manner through the TLR4/NF-κB pathway, culminating in a deeply immunosuppressive tumor microenvironment (26). Our findings, together with emerging evidence from other cancer types, establish the LPS/TLR4/NF-κB axis as a conserved pathway that shapes the immunosuppressive landscape across diverse tumors (36–38). Our study not only further validates the applicability of this mechanism in colorectal cancer models, but also provides new experimental evidence supporting the critical role of LPS-mediated TAM functional modulation in driving tumor progression.

The human anti-tumor immune response represents a precisely orchestrated, multi-tiered defense mechanism that integrates both innate and adaptive immune components through coordinated interactions (39). Within this sophisticated immunological network, the cGAS-STING pathway serves as a central regulatory hub (40). When macrophages phagocytose tumor-derived DNA within the tumor microenvironment, this triggers activation of the cGAS-STING signaling cascade, inducing robust type I interferon production (41–43). These interferons subsequently drive macrophage polarization toward an immunostimulatory M1 phenotype characterized by enhanced antigen presentation capabilities. Furthermore, this cascade facilitates the recruitment and activation of cytotoxic CD8+ T lymphocytes and natural killer (NK) cells, collectively establishing a potent anti-tumor immune surveillance system.

Based on this pivotal mechanism, STING agonists have been regarded as a highly promising therapeutic strategy for cancer treatment. However, clinical studies have demonstrated limited efficacy of STING agonist monotherapy, which may be attributed to their activation of negative feedback regulation (44). Our study elucidates a crucial underlying mechanism: LPS perturbs the precisely coordinated STING-mediated anti-tumor immune response by reducing tumor cell sensitivity to cytosolic DNA and elevating the activation threshold of the cGAS-STING pathway. These alterations collectively result in suppression of macrophage-mediated anti-tumor functions and induction of an immunosuppressive tumor microenvironment. These findings provide a compelling explanation for the suboptimal therapeutic efficacy observed with STING agonists in clinical settings.

More importantly, our study provides the first experimental evidence that the aminoglycoside antibiotic neomycin possesses dual anti-tumor mechanisms: specifically, it neutralizes LPS-mediated immunosuppression while synergizing with the STING agonist Cridanimod (CMA, 10-Carboxymethyl-9-acridanone) to significantly potentiate STING pathway activation (45).This pivotal finding not only clarifies how bacterial components regulate tumor immunity, but more critically, proposes an innovative combination strategy to address the clinical challenges of STING-targeted therapies, with substantial translational potential.

## Data Availability

The original contributions presented in the study are included in the article/supplementary material. Further inquiries can be directed to the corresponding author.
